# Awaiting accurate scientific evidence: Progression or “profiles” in bipolar disorder?

**DOI:** 10.1186/s40345-017-0087-3

**Published:** 2017-05-31

**Authors:** Diego J. Martino, Cecilia Samamé, Sergio A. Strejilevich

**Affiliations:** 1 Bipolar Disorder Program, Institute of Neurosciences, Favaloro University, Gurruchaga 2463, 1º C, 1425 Buenos Aires, Argentina; 2 National Council of Scientific and Technical Research (CONICET), Buenos Aires, Argentina; 3 Institute of Cognitive and Translational Neuroscience (INCyT), INECO Foundation, Favaloro University, Buenos Aires, Argentina

**Keywords:** Bipolar disorder, Response to treatment, Staging, Neuroprogression

## Abstract

This letter is written in response to a review recently published in the journal. The aim is to highlight a potential methodological limitation common to many studies comparing bipolar patients with few previous episodes versus those with multiple episodes, and in which the results are interpreted as indicating the longitudinal course of the illness.

Dear Editor,

We read with great interest the recently published review by Joyce et al. (2016) entitled “Is treatment for bipolar disorder more effective earlier in illness course? A comprehensive literature review”. After reviewing ten studies (eight research reports and two meta-analyses), the authors conclude that both psychological and pharmacological treatments earlier in the course of bipolar disorder (BD) are more effective than in the later stages in a range of clinical (i.e. symptomatic or relapse) and functional (i.e. vocational functioning or independent living) outcomes. They suggest that their findings could be explained by the hypothesis of neuroprogression and staging models proposed for BD, according to which there is a progression from at-risk to refractory and disabling presentations of the illness (Berk et al. 2007; Kapczinski et al. 2009).

In addition to the limitations described very accurately in the review, we would like to highlight an unconsidered aspect that could be critical for interpreting the results. BD is a very heterogeneous illness in terms of severity, with some patients experiencing a few recurrences and complete functional recovery throughout their lifespan and others showing multiple episodes and marked functional impairment despite treatment (Goodwin and Jamison 2007). In this review, as well as in neuroprogression and staging models, it is assumed that all patients with BD are equivalent at illness onset. However, based on the current evidence, it is difficult to rule out the possibility that at least part of the variability in terms of clinical severity, risk of recurrence, cognitive and functional impairments, and response to treatment would be intrinsic to each patient and present from the beginning of the illness (Martino et al. 2016). If this were the case, in a group of patients with few episodes (or at first episode) it would be likely that all the patients would be more equivalently represented than in a multi-episode group, which by definition would be biased towards the more severe forms of the illness (because patients with few episodes and benign clinical course are excluded). Then, even assuming a nonprogressive clinical course (and a similar response to treatment at any stage), patients with multiple episodes would be expected to display worse clinical and functional outcomes than patients with fewer episodes in response to a given treatment. Therefore, we suggest that the results of this type of methodological approach should not be considered as valid evidence of progressive resistance to treatment, because they could only mean that patients with more severe forms of the disorder are less responsive to treatment, which occurs with almost all diseases in medicine (Martino et al. 2016).

Joyce et al. (2016) proposed that a methodologically robust study design to answer the question of whether treatment is more effective earlier in illness course would be to sample treatment-naïve individuals with a first episode and multiple previous episodes of illness and compare treatment effectiveness between the groups. However, this approach would not solve the limitation set out above. In contrast, we think that any methodology used to test this hypothesis should try to ensure that the patient groups compared are as equivalent as possible with respect to the severity of their clinical course. For example, one could compare the effectiveness of a given treatment in patients with a first episode versus that found in patients with a second or third episode. Alternatively, it could be useful to compare the response to treatment of first-episode patients (of which a prospective follow-up of the later clinical course is available) with that of multiple-episode patients having a similar clinical course. Of course, the best design would be a longitudinal study in which to evaluate the effectiveness of a given therapeutic intervention along the successive episodes in the same patient. Unfortunately, none of these types of studies have been performed to date.

Clinical staging and neuroprogression proposed for BD are very attractive and potentially useful models (Berk et al. 2007; Kapczinski et al. 2009). They have been widely disseminated in the last decade and even included as part of the state of the art in a recent BD seminar (Grande et al. 2016). However, it should not be overlooked that they are hypotheses supported by several reviews such as that by Joyce et al. (2016) rather than empirical evidence derived from studies specifically designed to test them. Until further data are available, we should be extremely cautious with the interpretation of these reviews, as well as staging models, since they could only be describing the existence of subgroups of patients with different severity of their clinical course rather than the progression of the disorder at a particular point of time (Martino et al. 2016). Of course, in any case, these uncertainties do not contradict the clinical need for an early diagnosis and an accurate treatment in a disorder that is in itself disabling and potentially lethal.
